# Medical Examinations by State Boards

**Published:** 1883-06

**Authors:** 


### MEDICAL EXAMINATIONS BY STATE BOARDS.

The attention of the public, as well as of the profession of this city, has been lately attracted in the columns of the daily press, to the subject of the examination and licensing of practitioners of medicine by State boards. The discussion of the questions connected with this subject was originated by the proposition of the following resolution in the Chicago Medical Society:

"*Resolved*, That the public good would be promoted by the establishment of a State board of medical examiners, such board to be entirely separate and independent of all medical colleges; to have the exclusive right to grant license to practice medicine in the State of Illinois, leaving to medical colleges their function of teaching and conferring degrees, but obliging all who in future desire to enter upon practice, and who have not already received license to do so, to go before such board to prove their fitness; and that said board be required carefully to examine all applicants as to their moral, literary, and medical attainments, and only to confer a license on those who are well qualified in all these respects."

This resolution was finally, after discussion, adopted; and its mover immediately after offered the following, which in its turn also became the sense of the society:

"*Resolved*, That a committee of three be appointed by the chair to represent the Chicago Medical Society, and that they be instructed to confer with the Illinois State Board of Health relating to statements contained in the proceedings of its last meeting; and that this society respectfully requests said board to communicate to our committee any facts in the possession of the board that will enable the committee to prepare its report for the society."

The agitation of this important subject is timely and profitable. Some reform in existing methods is demanded, both by the profession and the public. The united opinion of the two is likely, at no distant date, to accomplish some actual results. It is time to ask, after due consideration of all sides of the question, what is the best and most practicable method of arriving at the desired end?

Whenever there are conflicting interests in a case at bar, it is commonly deemed prudent to compromise, if possible. Here there are interests at stake; grave interests of right and property, but in reality no interests which conflict. The highest interest of all concerned is best conserved by a single course. In deciding as to the proper course, however, it is neither safe nor wise to neglect any of the interests represented.

Thus, for example, the views of the teachers and representatives of the medical schools of the State should be fully ascertained and accorded weight before action be had in so important a case, lest that action be intemperate or partial, and thus fail of its desired end. Certainly these gentlemen are entitled to such consideration. In years, in learning, and in experience as both teachers and examiners in medicine, they have absolutely no superiors in the State of Illinois. Among the thousands practicing medicine in the State, it would be extremely difficult to select one hundred gentlemen who have done more to elevate the standards of their own ranks, and to contribute more largely to the respect entertained throughout the country at large for their contributions to scientific literature.

Nor is this all. The present professional position and fair prospects of the medical men of the Northwest are largely due to their influence. It is safe to say that over one-half of all the practitioners in the State were made such by their professional studies in Illinois schools of medicine. We cannot but express the conviction that it would be simply monstrous for the graduates of these schools, men who left their walls when the college curricula were admittedly less severe, and the requirements less rigid than at present, to favor any radical measures adverse to the common interests of those who admitted them to the ranks of medicine, and have since jealously guarded the professional reputation and name of the class with which they thus became identified. Looking at the question from this just and broad standpoint, there can be but one view taken of it. Decidedly, the examination and licensing of qualified practitioners should be conducted by a body of men not interested in their preparation for such an ordeal. We do not believe that there is a really respectable medical school in the country which will object to being relieved of this task, a no small addition to the really arduous labors of its teaching corps, always provided there is an equitable and general system of examinations in force, discriminating against no single institution, and rigorously enforcing its rules in the case of applicants from each and every college.

This is an important proviso. For example, it would be manifestly unfair and unjust to propose an examination for graduates of Illinois schools different from that imposed upon graduates of schools not situated in this State. If any discrimination were to be made, it should certainly favor those applying from our home schools. These home schools are entitled, the respectable ones among them at least, to a special consideration. Some of the gentlemen actively identified with their interests, have toiled incessantly for nearly half a century to establish them solidly, to enlarge their reputations, and to make this fourth city in size of the Union, a great medical educational center, not only of the Northwest, but of the country at large. One-quarter of a million of dollars of invested capital is the fruit of an incredible amount of self-sacrifice and victories won over opposition of the most senseless and ignorant character. Nearly one hundred thousand dollars are annually paid into their treasuries, by far the largest part of which is annually re-expended for apparatus and the expensive requirements of high-class medical teaching. The gentlemen engaged in this laborious effort to perfect and enlarge the American system of education in medicine handed down to us by our fathers in medicine, a system both unaided and unobstructed by legislation, though unquestionably and justly benefited in reputation, have not been rewarded by any such large pecuniary returns as would make them the objects of envy to those not similarly situated. The wealthy among them are few, far fewer than the professional men of wealth not enjoying such a connection. Many of them are financially in very moderate circumstances. What measure of success they have attained, is the reward of the self-sacrificing toil of years. These are considerations which the fair-minded will not ignore. If Illinois is to be so discriminated against by legislation that the young men of the Northwest, ambitious to enter the profession, find it necessary to pass by her doors and to seek their medical education in Massachusetts, or New York, or Philadelphia, let there be, we repeat, an impartial reviewing of the whole question before any irrevocable steps be taken. An unjust law usually defeats itself. Manifestly the only strictly fair and impartial system of examining and of licensing practitioners, would be one operating equally in all States of the Union, in New York as well as in Illinois, in Massachusetts as well as in Iowa.

A board of examiners, authorized to license for practice, and limited in its powers to this State, would not necessarily do all and be all that the dream of a *doctrinaire* might devise. Would there be a scramble for its membership in order to gratify the friends or the enemies of the several schools affected by its operation? Would it be insensible to the influences which have been powerful in the similarly constituted British Examining Boards? Creature of legislation, would it be superior to the corruption of American politics and politicians? Would it surpass the standards of the Illinois State Board of Health, which has, as a matter of fact, examined, passed and licensed under-graduates of Illinois medical colleges? Would it operate, as the last-named body has operated, as the fulcrum of a lever to elevate in public honor and esteem eclecticism and homœopathy, which have thus acquired a legal status and privileges? Would it refuse a license to the illiterate adventurer from a wretched little diploma-mill in Boston, and yet be powerless against the men in this city practicing under false names? Would it exact a fee for its examinations, and eke out a scanty legislative appropriation by subsistence of this sort? What would be the pay of its members? The United States Government lately offered but $2,500 *per annum* for some medical experts it needed. Can the State offer more than that to its examiners? Where are the men more just, better trained, wiser in science, and more skilled in medical examinations than those now doing this work, whose medical practice is so small that such a salary offered by the State is a temptation to abandon the former for the latter? Will such a board of examiners be mongrel in constitution, the irregulars disproportionately represented, thus inducing inferior students to graduate from the poorer schools possessing such disproportionate power in license? These are serious questions, and they must be answered. The respectable medical schools of this State would be indeed short-sighted if they did not welcome a just and equitable system of examination, but, stripped of their present powers and with the questions unanswered which are set forth above, they had better dispose of their property at once and invest it in something more respectable and profitable than mimicking a court fool to secure the favor of a prince.

Far better than a mongrel board would be a committee appointed by the State Medical Society of each sect, empowered by State law to examine the candidates of their own persuasion. Then, indeed, the State might license, and license only as thus advised, the physician and surgeon to practice and announce himself to the public only as a physician and surgeon; the homœopathist, only as such; the "physio-medical," the eclectic, *et id omne genus*, only as such.

But even under such a system as this, the poorest students would naturally seek the poorest schools, as offering them a cheaper, shorter, and readier pathway to the privileges of practice. Released from the control and influence of even a mongrel board, the sects would flourish most if the seal of the State were finally set in approval on the licenses furnished each. It is difficult to see how the best schools will fail to be the greatest sufferers under *any* law. They have indeed achieved, acting solely under the laws ensuring the survival of the fittest, in point of the number of students in attendance upon them, as well as in the matter of professional reputation and professional influence, an unquestioned supremacy. This is so well recognized, that their methods and manners as regards the public, are copied to the very letter by the smaller and inferior schools which aim to be their rivals.

The injustice of a law which operates unequally in different States of the Union, is so manifest that it scarcely requires comment. If a physician be such by virtue of the laws of the State of New York, has the State of Illinois power to disqualify him for practice? If she should attempt this, there can be but little question that an issue will be taken in some case for final presentation at the bar of the Supreme Court of the United States.

We have then a word for those who are interested in this matter—and every member of the profession should be so interested. Let the concurrence of the colleges be secured at the outset. The movement is timely, and may lead eventually to the most desirable results. If there be a medical school in the country, of respectable standing, which objects to the operation of a general system of examination and license, uniform throughout the whole country and impartial in operation, let that school bear the opprobrium which such an opposition will deserve. But if an effort is to be made (and there are indications that it will be made), to empower the Illinois Board of Health to examine and license the graduates of the medical schools of this State, such an effort will awaken the united opposition of the colleges and the large mass of reputable physicians in this State who are in affiliation with them. Before any such power shall be vested in that body, there will be a demand for its reconstruction, which will be loud and emphatic. When the Governor of the State of Illinois, recognizing the influence and numbers of the professional men of the city of Chicago, called together some of its representative members, many of them connected with the medical schools of the city, and requested them to nominate to him one of their number as a member of the State Board of Health, he gave weight to their opinions by appointing their nominee. Doubtless the same men can and will, if occasion arise, make their influence again felt. Before the Illinois State Board of Health is empowered to examine and license the graduates of the medical schools of this city, it will cease to be mongrel in character, or if not, will in its membership represent in due and just proportion the thousands of respectable practitioners in this State, as against the claims of homœopathists, eclectics, clergymen, lawyers, politicians, and decayed place-seekers of every description.

